# Interlaminar Bonding Properties on Cement Concrete Deck and Phosphorous Slag Asphalt Pavement

**DOI:** 10.3390/ma12091427

**Published:** 2019-05-01

**Authors:** Guoping Qian, Shunjun Li, Huanan Yu, Xiangbing Gong

**Affiliations:** 1 National Engineering Laboratory for Highway Maintenance Technology, School of Traffic and Transportation Engineering, Changsha University of Science & Technology, Changsha 410114, China; guopingqian@sina.com (G.Q.); xbgong@csust.edu.cn (X.G.); 2 School of Traffic and Transportation Engineering, Changsha University of Science & Technology, Changsha 410114, China; shunjun.li@stu.csust.edu.cn

**Keywords:** asphalt mixture, cement concrete deck, phosphorous slag, interlaminar bonding, shear strength

## Abstract

The slippage damage caused by weak interlaminar bonding between cement concrete deck and asphalt surface is a serious issue for bridge pavement. In order to evaluate the interlaminar bonding of cement concrete bridge deck and phosphorous slag (PS) asphalt pavement, the shear resistance properties of the bonding layer structure were studied through direct shear tests. The impact of PS as a substitute of asphalt mixture aggregate, interface characteristics, normal pressure, waterproof and cohesive layer types, temperature and shear rate on the interlaminar bonding properties were analyzed. The test results indicated that the interlaminar bonding of bridge deck pavement is improved after asphalt mixture fine aggregate was substituted with PS and PS powder, and the result indicated that the shear strength of grooved and aggregate-exposed interfaces is significantly higher than untreated interface, the PS micro-powder or anti-stripping agent can also improve the adhesion between layers when mixed into SBS asphalt. This study provided important theoretical and practical guidance for improving the shear stability of bridge deck pavement.

## 1. Introduction

The early damage, especially the slippage and upheaval of asphalt pavement over cement concrete bridge deck is a serious issue for bridge operations. When the bridge is under an extensive heavy vehicle load, large shear stress is generated inside the bridge deck pavement which caused uncertain shear failure surface; or serious shear damage and diseases are caused because the horizontal shear resistance between the pavement layer and bridge deck is weak [1]. Among the factors affecting the interlaminar bonding of bridge deck pavement structure, the mixture aggregate characteristic plays a significant role in that [2]. However, the lack of high-quality aggregates is very common in many areas, the mining of raw aggregate have cost many environmental problems, and the problem has become more and more serious with the acceleration of the infrastructure construction process.

Phosphorous slag (PS) is an industrial by-product produced in the process of phosphorus ore in a high-temperature environment. At present, the global discharge of wasted PS is about 12 to 15 million ton each year. The accumulation of a large amount of PS not only occupies the land but also seriously pollutes the environment [3]. Therefore, the effective utilization of PS in the asphalt pavement would beneficial the highway construction and the environment from all prospects [4].

Many pieces of research have been carried out on the engineering application of PS. Allahverdi, et al. [5] found that PS powder can significantly improve the quality of cement after mechanical activation and chemical catalysis. Zhao, et al. [6] demonstrated that PS with appropriate cement replacement dosage and specific area could improve the mechanical properties of sleeper concrete under steam curing. Xia, et al. [7] studied the crack resistance of PS concrete from the aspects of physical properties, hydration heat, shrinkage and creep and found that the crack resistance of PS concrete is approximate to, even to some extent better than, that of fly ash concrete. He, et al. [8] found that with the increase of granularity of PS additive, there are significant increases in the uniformity of particle sizes, slurry pH, and activity index, and the effects on cement paste are significantly mitigated. Jin [9] studied the influence of superfine PS as mineral filler on the long-term performance and durability of concrete. The results show that PS can improve the pore structure of concrete which is beneficial to the development of compressive strength and splitting tensile strength of concrete at a late age. Hamideh, et al. [10] predicted and optimized the compressive strength of PS cement at different ages (3, 7 and 28 days) based on the response surface method.

Qian, et al. [11] found that PS can be potentially used as an antistripping additive in asphalt mixture because the pH of the slag was alkaline and it was also hydrophobic and stable at high temperature. The viscoelasticity test also showed that PS filler improved the stiffness of asphalt. The mixture performance tests also indicated that PS filler significantly increases the resistance of HMA to rutting and moisture damage. Qian, et al. [12] further analyzed the influence of surface modified PS powder as a modifier on the mechanism of asphalt and asphalt mixture and found that TM-P modified PS powder can enhance the compatibility with asphalt, which improves the antiaging, rutting resistance and water damage resistance of asphalt mixture. Sheng, et al. [13] studied the effect of PS powder as mineral filler on the rheological properties of asphalt binder and the properties of asphalt mixture and found that it increased the binder viscosity resulting in enhanced mixture rutting resistance. In order to study the rutting and fatigue damage of asphalt pavement, Bazzaz, et al. [14,15] proposed a straightforward procedure to characterize the nonlinear viscoelastic response of asphalt concrete materials. 

In addition, there are many pieces of research that have been carried out on asphalt pavement structure of cement concrete bridge deck which provided meaningful guidance on this research. Wang, et al. [16] have studied the interface shear characteristics between the asphalt pavement structure and the concrete bridge deck pavement and found that the shear strength of SBS modified asphalt pavement over concrete bridge deck is slightly greater than that of crumb rubber modified asphalt pavement. Li, et al. [17] studied the interlaminar failure modes and mechanisms of rubber powder modified asphalt, SBS modified asphalt and epoxy resin adhesive as waterproof bonding materials and found that the shear strength is greatly affected by the thickness of the waterproof adhesive layer. Liu, et al. [18] studied the bonding performance of waterproof bonding layer between the concrete bridge deck and asphalt mixture pavement by a lab test, field temperature monitoring and finite element method (FEM). The test results show that the safety factor (strength/stress) decreases significantly with increasing environmental temperatures. Sheng, et al. [19] established a simplified formula for calculating the extreme temperature (maximum and minimum temperature) stress of bridge deck pavement structures. The results have shown a strong linear correlation between the bridge deck pavement maximum principal stress and the elastic modulus. 

He, et al. [20] proposed typical structural types of cement concrete bridge deck pavement based on waterproof cohesive layer material test and bridge deck pavement composite structure test. Jia [21] proposed using the interlayer shear test as a method to study the shear performance of the interlayer structure layer of bridge deck pavement. And have proposed design method and design standard of asphalt mixture based on that. Ren, et al. [22] analyzed the influence of chip-sprinkling interlayer treatment technology on the shear resistance of cement concrete bridge deck asphalt pavement layers, and recommended the optimal chip-sprinkling technique parameters. Xu, et al. [23] evaluated the improvement effect of aggregate-exposed interface on the stability of bridge deck pavement structure by direct shear test and pull-out test. They found that exposed-aggregate showed better shear performance than other interfacial treatment methods under various positive pressures. Liu, et al. [24] proposed the environmental simulation bubble test and used MatchID-3D structural deformation analysis system to measure bubble deformation, and studied the deformation characteristics and mechanism of bubbles in bridge deck waterproofing membrane. It found that the test temperature, initial debonding aperture, and water have great influences on the performance of bridge deck pavement. Lee, et al. [25] have studied the feasibility of reducing early temperature shrinkage crack and dry shrinkage crack of low melting point concrete with shrinkage reducing agent. And confirmed the durability can be increased without affecting other properties by adding a shrinkage reducing agent.

Although there are many pieces of research on the application of PS in HMA, research on the interlaminar shear resistance of cement concrete bridge deck PS asphalt pavement is relatively limited. In this paper, interlaminar shear strength is used as evaluation indexes, and the bonding performance of the interlayer structure is evaluated through the direct shear test with normal pressure. The impact of PS as a substitute for the asphalt mixture aggregate, interface types, normal pressure levels, waterproof bonding layer types, temperature and shear rate on interlaminar bonding shear performance are analyzed.

## 2. Experimental Plan

### 2.1. Mixture Design

**Figure 1:**
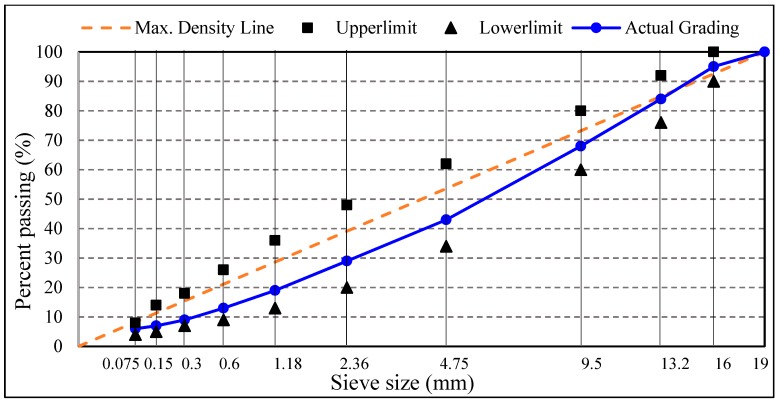
The gradation of dense-graded AC-16C asphalt mixtures.

In this research, “Shell” SBS modified asphalt binder was used in the design of pavement surface layer and an interlaminar bonding layer. The conventional test of asphalt and asphalt mixture was following the procedures of “standard test methods for bitumen and bituminous mixture for highway engineering” (JTG E20-2011) [26]. The conventional asphalt binder test results are shown in Table 1, as shown in the table that the asphalt binder satisfied the specification requirement. The asphalt mixture was designed following the steps of “technical specification for construction of highway asphalt pavement” (JTG F40-2017) [27], the aggregate used was limestone, the filler was limestone mineral powder, the optimum asphalt content was 5.4%, and the pavement mixture gradation was widely-used AC-16C. The gradation curve of the asphalt mixture is shown in Figure 1 and the design and volumetric parameters of asphalt mixture are shown in Table 2. In order to evaluate the influence of PS on the shear resistance of asphalt mixture, the fine limestone aggregate of 0.075 mm–4.75 mm was replaced with PS and PS powder for comparative tests.

### 2.2. Experiment Design

**Figure 2:**
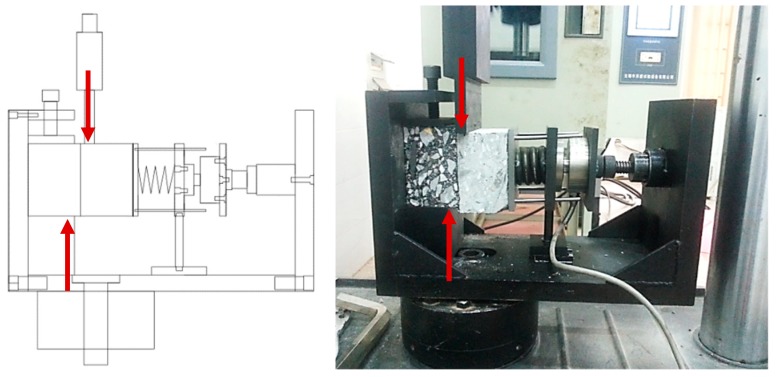
Direct shear test design and test device.

The interlaminar shear resistance of bridge deck pavement specimens was studied by direct shear test under normal pressure. The specimens used were prismatic specimens of 80 mm × 80 mm × 100 mm. The test design and test equipment (Material Testing Systems—MTS 810, USA) are shown in Figure 2.

Four duplicate specimens were prepared for each test, and the test results were illustrated with the average of four specimens. The test specimens preparation steps were as follows:

(1) Cement concrete test specimens were formed indoor following the construction process with a size of 300 mm × 300 mm × 50 mm. Then, specimens were placed in the standard curing room for curing of 28 days, and the cement concrete panels were prepared with three types of interface: untreated (without any surface treatment), grooved and aggregate-exposed, the sample preparation process of the grooved interface and aggregate-exposed interface were shown below:

*(a) Grooved interface.* The grooves were notched according to the pavement anti-slide requirements in the technique guidelines for construction of highway cement concrete pavement [28]. The grooves were notched in a depth of 2–4 mm, in a width of 3–5 mm, and a groove spacing of about 15 mm.

*(b) Aggregate-exposed interface.* The first step prepares the aggregate-exposed concrete was to spray retarder on the surface of the cement concrete layer after paving, which delayed the setting and hydration of the surface mortar layer but did not affect the normal setting and hydration of the main body. After the main-body concrete reaches a certain strength, the surface laitance was washed out to expose part of coarse aggregate.

**Figure 3:**
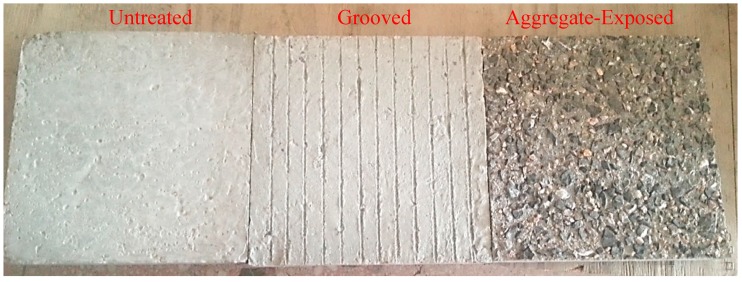
Cement concert slabs with three interface types.

After curing, the cement concrete slabs of the three interface types are shown in Figure 3, which displays as the untreated interface, the grooved interface and the aggregated-exposed interface from left to right.

**Figure 4:**
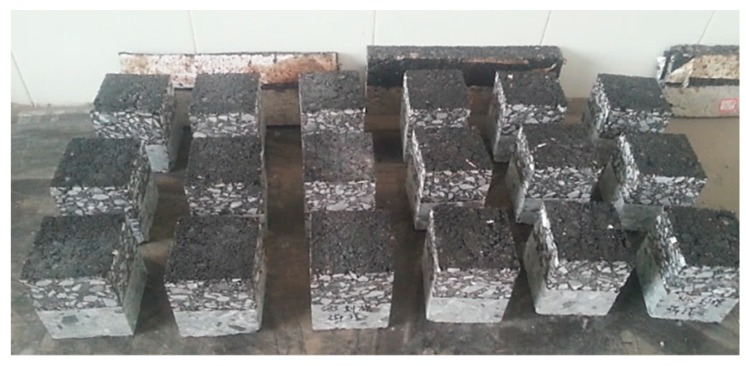
Specimens for the direct shear test with different interfacial treatment.

(2) After the cement concrete slabs were cured, the surface of each type was coated with three different types of interlayer bonding materials separately, which included SBS modified asphalt, SBS modified asphalt mixed with PS micro-powder, and SBS modified asphalt mixed with surfactant (anti-stripping agent). The content of PS micro-powder and anti-stripping agent was 10% and 0.4% of the mass of asphalt, respectively. As the surface area of each type of interface was different, in order to make sure that all surfaces were coated well, the dosage of waterproof cohesive bonding material for untreated, grooved and aggregate-exposed interfaces was 1.0 kg/m^2^, 1.2 kg/m^2^ and 1.5 kg/m^2^ respectively. 

(3) Then, put the cement concrete specimen into a 300 mm × 300 mm × 100 mm rutting plate test mold, and poured the mixed asphalt mixture over it and applied the rutting wheel to compact it into the desired compaction level. Finally, the composite specimens were cut into 80 mm × 80 mm × 100 mm small prism specimens. The specimens with different interfacial treatment are shown in Figure 4.

For the direct shear test, the shear strength corresponding to the peak value of the load-displacement curve is the shear strength of the interface. The calculation method is shown in Equation (1):

*τ* = *P*/*S*. (1)

In which: *τ* is the interlaminar interface shear strength (MPa); *P* is the peak value of shear load in the direct shear test (kN); *S* is the interfacial area (mm^2^).

**Figure 5:**
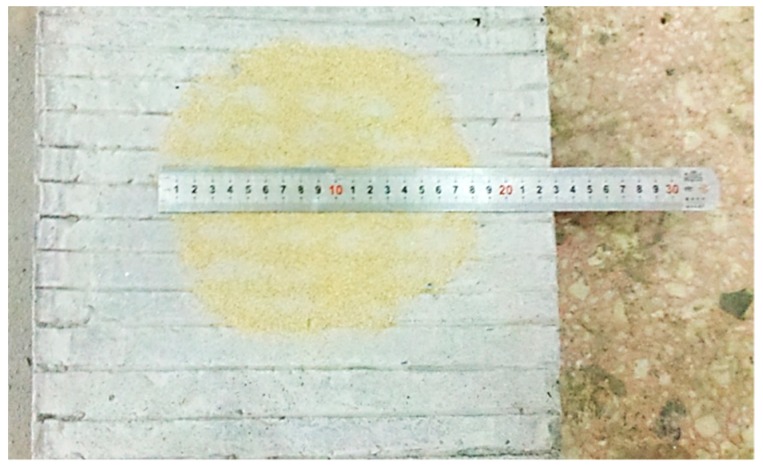
Method to measure the texture depth.

As the surface roughness of cement concrete slabs with three interface types of untreated, grooved and aggregate-exposed was different, the roughness characteristics were evaluated by the texture depth (*TD*) index. The *TD* was measured by sand spreading method following the field test methods of subgrade and pavement for highway engineering (JTG E60-2008) [29] which was described below: Firstly, spread the standard sand on the cement slab into a circle, then scrape the surface of the standard sand with a scraper, measure the diameters of the two vertical directions of the circle with a ruler, then brush the standard sand on the cement board with a clean brush to weigh the quality. The *TD* of the cement board surface can be calculated by Equation (2). The determination of the *TD* of the cement slab is shown in Figure 5. The *TD* measurement results for untreated, grooved and aggregate-exposed interfaces are 0.59 mm, 1.67 mm and 4.43 mm respectively.

(2) $${TD\;=\;4V/\pi d}^{2}.$$

In which, *V* is the sand volume to filling the uneven part under the measuring circle (mm^3^), *d* is the diameter of the measuring circle (mm).

### 2.3. Experimental Schematic

In order to evaluate the impact of PS as a substitute of asphalt mixture aggregate, waterproof and cohesive layer material type, bridge deck interface treatment, normal pressure, test temperature and loading rate on the interlaminar bonding behavior of bridge deck pavement, the following tests were conducted:

(1) **Impact of PS as a substitute of asphalt mixture aggregate.** The asphalt mixture of AC-16C was used to compare the interlaminar bonding of PS as a substitute of asphalt mixture aggregate: one is named as limestone asphalt mixture in which the asphalt mixture aggregate was limestone and filler and the other was named as PS asphalt mixture in which the fine limestone aggregate of 0.075 mm to 4.75 mm and fillers were replaced by PS and PS powder in equal amounts.
(2) **Impact of waterproof and cohesive layer material type.** In order to compare the interlaminar bonding behavior of different bonding layer materials, SBS modified asphalt, SBS modified asphalt mixed with PS micro-powder and SBS modified asphalt mixed with an anti-stripping agent were selected for comparison.
(3) **Impact of bridge deck interface treatment.** In order to analyze the impact of different interface conditions on the shear resistance of interlayer, the bonding characteristics of untreated, grooved, and aggregate-exposed interfaces were evaluated.
(4) **Impact of normal pressure.** In order to study the influence of normal pressure on interlaminar shear strength, the test with normal pressures of 0 MPa, 0.3 MPa, 0.5 MPa and 0.7 MPa were conducted and compared respectively.
(5) **Impact of temperature.** The test temperature has a significant effect on the interlaminar shear strength. In order to obtain the impact of the test temperature on the interlaminar shear strength, five test temperatures of 25 °C, 40 °C, 50 °C, 60 °C and 70 °C were selected to conduct comparison tests.
(6) **Impact of shear rate.** In order to simulate the effect of different driving speeds on the interlaminar shear performance, five different shear loading rates of 1 mm/min, 5 mm/min, 10 mm/min, 20 mm/min and 50 mm/min were selected to conduct comparison tests.

## 3. Results and Discussions

### 3.1. Impact of PS as Asphalt Mixture Aggregate Substitute

**Figure 6:**
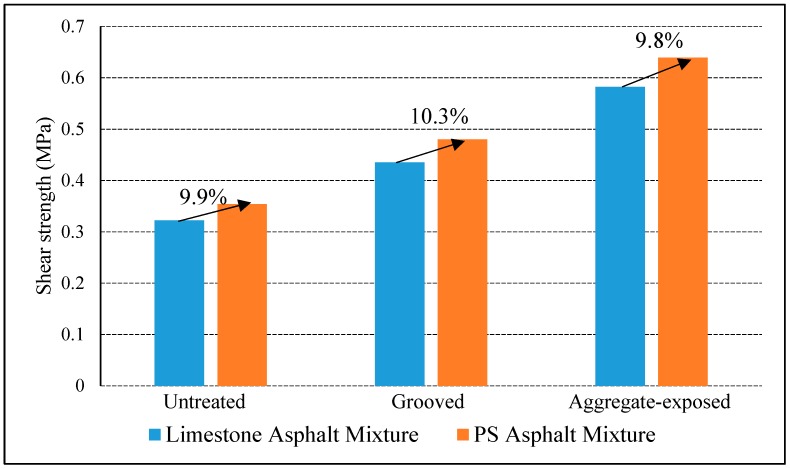
Impact of aggregate and interlayer treatment on the interlaminar bonding.

The direct shear strength between concrete deck and asphalt pavement was measured to evaluate the impact of PS as limestone substitute on the interlaminar bonding performance. The SBS modified asphalt was used in the interlaminar bonding layer, and the test temperature was 60 °C. In order to ensure the specimen had a constant test temperature, the specimens were put into the environmental chamber for 4 h before the test, the shear rate was 10 mm/min, the vertical pressure was 0.5 Mpa. The three different interface types of untreated, grooved and aggregate-exposed were selected for comparative study. The test results are shown in Table 3. The comparison figure is shown in Figure 6.

As shown in Table 3 and Figure 6, the interlaminar shear strength corresponding to the PS asphalt mixture is higher than that of limestone asphalt mixture for all three kinds of interface types, and the increasement corresponding to untreated, grooved and aggregate-exposed interfaces were 9.9%, 10.3% and 9.8% respectively. The main reason is that PS is alkaline and have a larger specific surface area, so it shows better adhesion to asphalt binder compared with limestone powder. As a result, the bonding effect between PS asphalt mixture and SBS modified asphalt material is improved, the shear strength and interlaminar stability of the bridge deck and asphalt pavement are improved.

### 3.2. Impact of Interfacial Surface on the Interlaminar Bonding

In order to study the influence of surface treatment on the shear resistance of the bridge deck, three kinds of interface types were evaluated in this paper, which including untreated interface, the grooved interface, and aggregate-exposed interface. Indoor direct shear tests were carried out on bridge deck pavement composed of two kinds of pavement materials: PS asphalt mixture and limestone asphalt mixture. The interlaminar bond coating materials were SBS modified asphalt, the test temperature was 60 °C, the shear rate was 10 mm/min, and the normal pressure was 0.5 MPa. The test results were also shown in Table 3 and Figure 6.

From Figure 6, it can be seen that both the shear strength of PS asphalt mixture and the limestone asphalt mixture shows the same relation for all three interface types: aggregate-exposed interface > grooved interface > untreated interface. When the paving layer is a limestone asphalt mixture, the shear strength of the grooved interface and the aggregate-exposed interface were increased by 35.1% and 80.7% respectively compared with the original untreated interface. In addition, for the PS asphalt mixture, the shear strength of the grooved interface and the aggregate-exposed interface were increased by 44.1% and 80.5% respectively compared with the original interface. 

**Figure 7:**
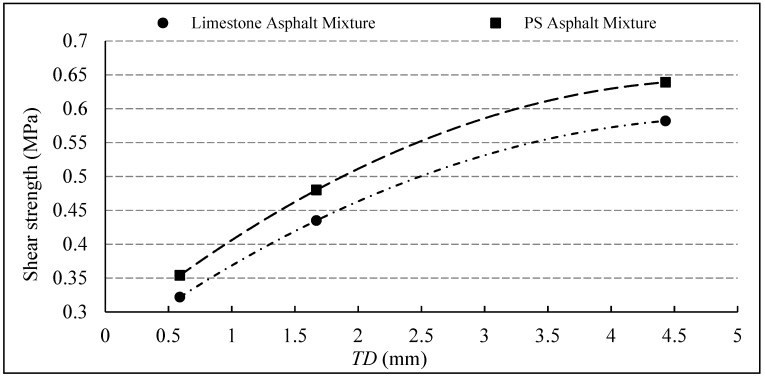
Impact of texture depth on the interlaminar bonding.

As mentioned earlier, the *TD* size of the three interface types is sorted as aggregate-exposed interface > grooved interface > untreated interface. The relation of *TD* on the shear strength of the composite structure is shown in Figure 7. The results showed that the shear strength closely related to the surface textural of the bridge deck, the rougher the texture of the deck surface and the greater the value of *TD*, the greater the shear strength of the corresponding deck pavement structure.

### 3.3. Impact of Waterproof and Cohesive Layer Material Type

**Figure 8:**
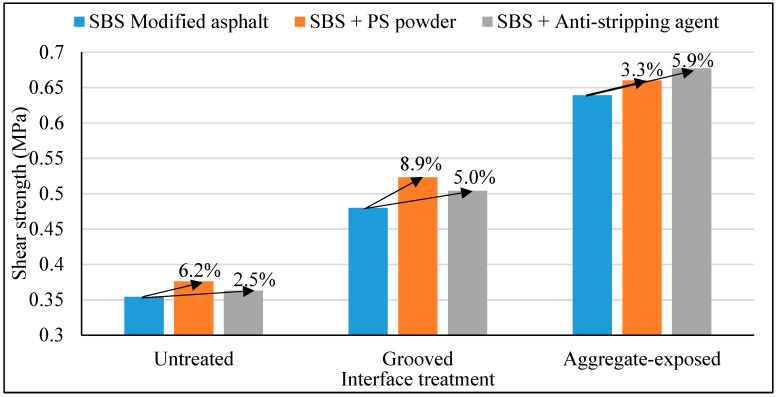
Interlaminar shear strength of different waterproof and cohesive materials.

In order to compare and study the influence of waterproof and cohesive coating material on the interlaminar shear resistance and to improve the interlaminar stability of bridge deck pavement, the interlaminar shear tests of SBS modified asphalt, SBS modified asphalt with PS micro-powder, and SBS modified asphalt with surfactant (anti-stripping agent) were carried out. The interlaminar shear test was conducted with three interface types mentioned above respectively. The PS asphalt mixture was used as a pavement layer. The test temperature was 60 °C, the shear rate was 10 mm/min, and the vertical pressure was 0.5 MPa. The test results are as shown in Table 4 and the comparative figure is shown in Figure 8.

As can be seen from Table 4 and Figure 8, For the original untreated interface, the shear strength increase by 6.2% and 2.5%, the grooved interface increased by 8.9% and 5.0% and the aggregate-exposed interface increased by 3.3% and 5.9%, respectively. The results show that the interlaminar shear strength of SBS asphalt with PS powder and SBS asphalt with anti-stripping agent were higher than that of SBS asphalt. The reason is that the surface of the grooves and exposed coarse aggregate increased the adhesion ability between the bridge deck and asphalt pavement, therefore, the contribution of the PS powder or anti-stripping agent on the interlayer shear stress of treated interface is greater than that of the untreated interface.

### 3.4. Impact of Normal Pressure

**Figure 9:**
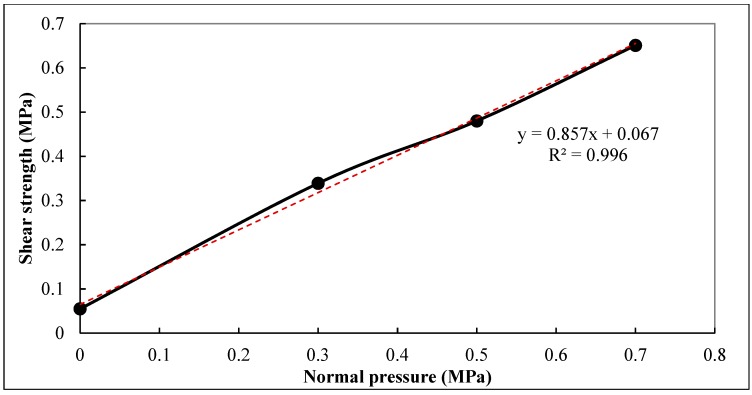
Relationship between normal pressure and interlaminar shear strength.

The mechanical properties of bridge deck pavement are greatly influenced by the grade of vehicle load. As the normal pressure at the interface of pavement and bridge deck is below 0.7 MPa at most cases, therefore, the tested normal pressure for the interlaminar bonding properties was in the range of 0 MPa to 0.7 MPa. In order to better study the impact of different vehicle loads on the shear stability of deck pavement, different normal pressures of 0 MPa, 0.3 MPa, 0.5 MPa and 0.7 MPa were selected respectively to conduct the direct shear test. SBS modified asphalt was used as a waterproof and cohesive layer and PS asphalt mixture was used as a bridge deck surface layer. The experimental temperature was 60 °C and the shear rate was 10 mm/min. The results of shear tests were shown in Table 5 and Figure 9.

Although the factors affecting the interlaminar bonding properties are very complicated, as it can be seen from Figure 9, it is reasonable to assume it satisfied the Mohr-Coulomb strength theory when the normal pressure was below 0.7 MPa, in which the interlaminar shear strength has a linear relation with the normal pressure. In addition, based on the Mohr-Coulomb strength theory, the interlaminar shear failure will not occur if the interlaminar shear stress caused by vehicle load satisfied the following equation:

(3) $${\tau \;<\;c\;+\;\sigma }_{z}tg\varphi$$

where *c* is the cohesion between asphalt pavement and cement deck, *σ_z_* is the normal compressive stress on the shear surface, and *σ_z_tgϕ* is the friction between the rough surface texture structure of bridge deck and the asphalt mixture of pavement. The linear regression equation of the interlaminar shear strength and the corresponding cohesive force *c* and internal friction angle *ϕ* of the grooved interface under different normal pressures are shown below.

(4) $$\tau \;=\;0.067\;+\;0.857\sigma \;(R^{2}\;=0.996).$$

In which, the cohesive strength of *c =* 0.067 MPa, and the internal friction angle *ϕ* = 40.6°.

### 3.5. Impact of Temperature on Interlaminar Shear Strength

As the interlayer bonding material of bridge deck pavement is usually viscoelastic material, the bonding properties are sensitive to temperature change, therefore, the bridge deck pavement tended to occur interlaminar shear deformation due to the decrease of bond performance at summer high-temperature conditions. 

In order to study the influence of temperature on interlaminar shear strength of PS asphalt mixture, the interlaminar shear tests were carried out at 25 °C, 40 °C, 50 °C, 60 °C and 70 °C respectively. In order to ensure the temperature field of the specimen was uniform, the specimens were placed in the environmental chamber for 4 h before the test. The interlaminar bonding material was SBS modified asphalt, and the interface was grooves treated. The experimental interlaminar shear rate was 10mm/min and the normal pressure was 0.5 MPa. The test results are shown in Table 6.

**Figure 10:**
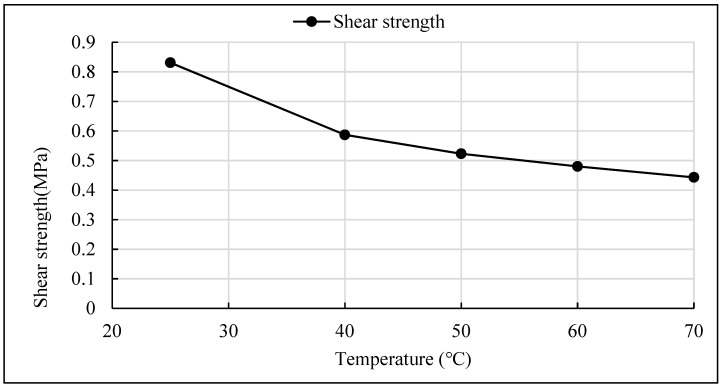
Relation of interlaminar shear strength with temperature.

As it can be seen from Figure 10, the influence of temperature on the interlaminar shear stress of PS asphalt pavement is very obvious, the interlaminar shear strength decreases gradually with the increase of temperature, and the influence slope varies in a different temperature range. It can be calculated from the diagram that the interlaminar shear strength decreased by 29.4% when the temperature increased from 25 °C to 40 °C, the shear strength decreased by 10.9% when the temperature raised from 40 °C to 50 °C, the interlaminar shear strength decreased by 8.9% when the temperature raised from 50 °C to 60 °C, and the interlaminar shear strength decreased by 7.7% when the temperature raised from 60 °C to 70 °C. From the experimental data, it can be seen that the interlaminar shear strength at 60 °C is only 57.7% of that at 25 °C, this would explain the reason why the interlaminar shear failure of bridge deck pavement occurs mostly in the high-temperature season.

### 3.6. Impact of Shear Rate on Interlaminar Shear Strength

In order to study the influence of different driving speed on the shear stress of PS asphalt pavement, various shear rates of 1 mm/min, 5 mm/min, 10 mm/min, 20 mm/min and 50 mm/min were used to simulate the different driving speeds. The grooved interface was used in these tests, the waterproof and cohesive material was SBS modified asphalt. The test temperature was 60 °C and the normal pressure was 0.5 Mpa. The test results are shown in Table 7 and Figure 10.

**Figure 11:**
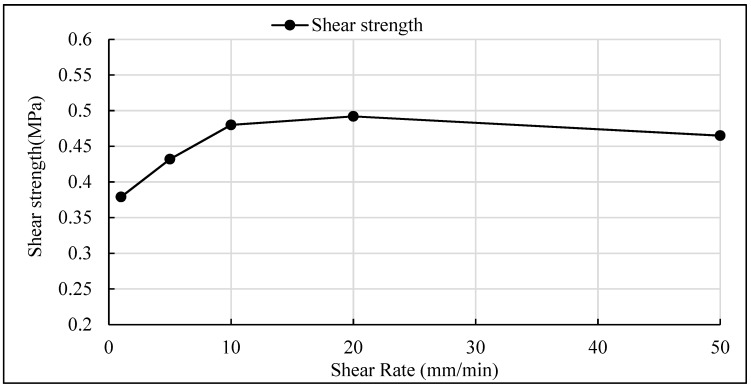
Relationship between shear rate and interlaminar shear strength.

As shown in Figure 11, the shear rate had a significant effect on the shear strength between layers, and the shear strength increased with the increase of shear rate when the shear rate was below 10mm/min, and when the shear rate increased from 1 mm/min to 10 mm/min, the effect of loading rate on shear strength was especially significant. As it can see from that test data that the shear strength of 10 mm/min was 26.6% higher than that of 1 mm/min. As the loading rate continues to increase, the test curve gradually tends to smooth and stable, that is, when the loading rate was large, the influence of loading rate on the interlaminar shear strength became smaller. This also shows that the vehicle’s damage to the pavement of the bridge deck at high speed was less than at lower speed.

## 4. Conclusions

This research evaluated the factors impacting the interlayer bonding and shear resistance of cement concrete bridge asphalt pavement, the comparatively tests including PS as a substitute of fine aggregate for asphalt mixture, interface characteristics, normal pressure, waterproof and cohesive layer types, temperature and shear rate. The following conclusions can be drawn:

(1) After the substitution of limestone aggregate between the size of 0.075 mm and 4.75 mm in the asphalt mixture with an equal amount of PS and PS powder, the bonding characteristics between surface asphalt mixture and bridge deck were improved, the interlaminar shear resistance and interlaminar stability were increased.
(2) The interfacial treatment of the bridge deck had a significant effect on the shear resistance of the deck. The rougher the surface texture and the greater the TD of the surface, the greater the shear resistance of the deck. The result indicated the aggregate-exposed treatment could increase the shear strength up to 80% compared with the untreated interface. It was an effective way to improve the shear resistance of bridge deck pavement by grooved or aggregate-exposed treatment.
(3) The impact of the test conditions on the interlaminar shear strength is significant. The interlaminar shear resistance increases with the increase of normal pressure, and the shear strength decreases gradually with the increase of test temperature. The interlaminar shear strength increases with the increase of shear rate in a certain range and tends to be stable when the shear rate is getting higher than 10 mm/min.

Overall, the bonding performance of bridge deck pavement can be improved by replacing the limestone aggregate and filler of 0.075–4.75 mm in asphalt mixture with PS and PS powder respectively. The rougher the deck surface is, the better the bonding strength between layers will be. Deck surfaces treated with the grooved or aggregate-exposed method can significantly improve the interlaminar bonding performance. Both PS powder and anti-stripping agent can improve the adhesion performance of SBS modified asphalt as a bonding layer.

## Figures and Tables

**Table 1:** Technical indexes of (Styrene-Butadiene-Styrene) SBS modified asphalt binder.

Properties		Test Value	Technical Index
Penetration at 25 °C, 100 g, 5 s (0.1 mm)		51.5	30-60
Ductility at 5 °C, 5 cm/min (cm)		28.5	≥20
Softening Point (°C)		87	≥60
After RTFOT	Weight Change (%)	−0.053	≤± 1.0
	Penetration Ratio (%)	80	≥65
	Residual Ductility at 5 °C, 5 cm/min	16	≥15

**Table 2:** Marshall test results of SBS modified asphalt.

Asphalt Content (%)	Void Ratio (%)	VMA* (%)	VFA* (%)	Stability (kN)	Flow Value (mm)
5.4	4.7	15.2	69	16.3	31.4

* VMA = Voids in Mineral Aggregate, VFA = Voids Filled with Asphalt.

**Table 3:** Impact of aggregate and interlayer treatment on the interlaminar bonding.

Aggregate Type	Interface Type	Max Shear Force (kN)	Shear Strength (MPa)
Limestone Asphalt Mixture	Untreated	2.061	0.322
	Grooved	2.784	0.435
	Aggregate-exposed	3.725	0.582
PS Asphalt Mixture	Untreated	2.266	0.354
	Grooved	3.072	0.480
	Aggregate-exposed	4.089	0.639

**Table 4:** Interlaminar shear strength of different waterproof and cohesive materials.

Bonding Material	Interface Type	Max Shear Force (kN)	Shear Strength (MPa)
SBS asphalt	Untreated	2.266	0.354
	Grooved	3.027	0.480
	Aggregate-exposed	4.089	0.639
SBS asphalt with PS Micro-powder	Untreated	2.406	0.376
	Grooved	3.347	0.523
	Aggregate-exposed	4.224	0.660
SBS asphalt with anti-stripping agent	Untreated	2.323	0.363
	Grooved	3.226	0.504
	Aggregate-exposed	4.333	0.677

**Table 5:** Test results of interlaminar shear strength under different normal pressures.

Pavement Mixture	Normal Pressure (MPa)	Max Shear Force (kN)	Shear Strength (MPa)
PS asphalt mixture	0	0.352	0.055
	0.3	2.169	0.339
	0.5	3.072	0.480
	0.7	4.166	0.651

**Table 6:** Results of interlaminar shear tests at different temperatures.

Mixture Type	Temperature (°C)	Maximum Shear (kN)	Shear Strength (MPa)
PS asphalt mixture	25	5.318	0.831
	40	3.757	0.587
	50	3.347	0.523
	60	3.027	0.480
	70	2.835	0.443

**Table 7:** Results of interlaminar shear strength tests at different shear rates.

Pavement Mixture	Shear Rate (mm/min)	Max Shear Force (kN)	Shear Strength (MPa)
PS asphalt mixture	1	2.425	0.379
	5	2.765	0.432
	10	3.072	0.480
	20	3.149	0.492
	50	2.976	0.465
